# ChatGPT's Performance on the Orthopaedic In-Training Examination (OITE): No Better Than a PGY3 Resident?

**DOI:** 10.7759/cureus.95253

**Published:** 2025-10-23

**Authors:** Karan Goswami, Neil Zhao, Temitope A Ayodele, Chad A Krueger, James Purtill, Alexander Vaccaro, Pedro K Beredjiklian

**Affiliations:** 1 Department of Orthopaedic Surgery, Rothman Orthopaedic Institute, Philadelphia, USA; 2 Department of Internal Medicine, Thomas Jefferson University Hospital, Philadelphia, USA; 3 Department of Orthopaedics, Zucker School of Medicine, Hempstead, USA

**Keywords:** chatgpt, in-training examination, oite, orthopaedic in-training exam, orthopaedic residency, orthopaedics

## Abstract

Background

Orthopaedic In-Training Examination (OITE) performance is an important metric for assessing resident knowledge and directing education. ChatGPT (OpenAI, San Francisco, California, United States) is a novel artificial intelligence (AI) large language model (LLM) with the ability to emulate human conversation and provide access to large pools of knowledge. Recent reports using ChatGPT have achieved a passing performance on medical licensing (United States Medical Licensing Examination (USMLE)) and legal examinations. Whether ChatGPT can successfully complete the OITE and be used as a didactic component in the education of residents remains unknown. This study was thus conceived to compare ChatGPT OITE performance versus local and national resident performance over the last five years.

Purpose

As ChatGPT and other LLMs grow in popularity, they are more commonly used by orthopaedic trainees to aid information acquisition and didactic learning. This study was conducted to elucidate the accuracy with which LLMs can answer questions on the OITE, a standardized knowledge and decision-making exam that acts as a benchmark for all orthopaedic trainees. This will help evaluate the current utility of this technology to orthopaedic trainees as a didactic tool throughout training.

Patients and methods

ChatGPT was provided 200 (10 sets of 20) randomly chosen questions from OITE years 2018 to 2022. All images without direct links present in the selected OITE questions were uploaded to an image hosting service, and links were provided alongside the corresponding question text when entered into ChatGPT. The primary outcome of interest was the percentage of correct responses. ChatGPT's performance was compared against institutional resident averages, as well as national orthopaedic resident averages, for each PGY class. Statistical synthesis comprised the one-sample Wilcoxon test and the Kruskal-Wallis test with the Dunn-Sidak correction.

Results

ChatGPT underperformed all PGY year national averages of allopathic/Accreditation Council for Graduate Medical Education (ACGME)-accredited orthopaedic surgery residents (p<0.01). Local institution PGY3 (p=0.0444), PGY4 (p=0.0045), and PGY5 (p=0.0004) resident classes also performed significantly better on the OITE. ChatGPT performed best on the 2021 exam (47.3%) and worst on the 2020 exam (35.3%). Overall ChatGPT performance for all five years was not significantly different (p>0.05).

Conclusions

ChatGPT scored lower but statistically equivalent to PGY1-2 orthopaedic surgery residents at our institution, although performance was lower than national PGY1-5 resident averages on the 2018-2022 OITEs. This performance is likely to improve in future iterations of ChatGPT as this remains a text-based language tool, not yet validated for image interpretation. Future generative language applications of ChatGPT are broad-ranging for continuing education and the assessment of residents-in-training.

Clinical relevance

As ChatGPT continues to gain popularity, it will inevitably be used by orthopaedic trainees in preparation for the OITE and future board examinations. To our knowledge, this study is the first of its kind to evaluate ChatGPT's performance on the annual OITE, providing insight into its current accuracy and limitations. These findings help clarify its potential role as an adjunctive tool in resident education and future orthopaedic training.

## Introduction

ChatGPT (OpenAI, San Francisco, California, United States) is a large language model (LLM) and machine learning system that autonomously learns from data and can produce sophisticated and seemingly intelligent writing after training on a massive text dataset [1]. It is the latest iteration of a series of such models, which was released to the public on November 30, 2022, by OpenAI. ChatGPT, formally known as Generative Pre-trained Transformer 3, is designed to learn language patterns by processing written text to generate a coherent response. It has been specialized for text generation, summarizing, classifying, completing, and responding to text, as well as writing code [2].

Recently, ChatGPT has generated much popular excitement and controversy due to its performance on medical licensing, legal qualification, and business administration examinations [3-5]. For example, ChatGPT was tested on the final examination for the MBA program at the University of Pennsylvania-Wharton School of Business and achieved a B-/B grade [3]. Additionally, ChatGPT was able to perform at a passing rate for two subsections of the "Bar" legal practice licensing examination, with its top 2 and 3 responses correct 71% and 88% of the time [4]. Furthermore, ChatGPT was tested on the United States Medical Licensing Examination (USMLE) (Step I, Step II CK, Step III) and achieved a passing score that typically reflects a medical student's culmination of knowledge before moving on to residency training [5]. Despite the promising performance of ChatGPT, there is some concern about the use of this technology for the potential pitfall in the accuracy of recall. According to the founders of OpenAI, since GPT-3 was trained using the internet and data from many different sources, there is no guarantee that outputs are accurate or truthful [6]. Additionally, as an autoregressive language model, once ChatGPT has started an incorrect prediction, it is unable to go back and correct it. Instead, it continues to predict each word based on the preceding words [7]. In other words, ChatGPT is capable of generating very reasonable-sounding but very incorrect answers.

ChatGPT has yet to be tested at a higher graduate-level medical examination, such as the Orthopaedic In-Training Examination (OITE). The OITE is an approximately 275-item, multiple-choice, computer-based examination that covers 10 subspecialty content domains, representative of the established principles, conventional procedures, and treatment modalities in orthopaedic surgery [8,9]. In 2021, it was administered to 4,987 residents across 231 orthopaedic residency programs [9]. The examination has evolved to a set of common items with the American Board of Orthopedic Surgery (ABOS) Part I certifying examination and serves as a rough benchmark to help guide education and board exam preparation for resident physicians [10].

This study aimed to evaluate ChatGPT's performance on the OITE, which is intended to assess the competence and knowledge base of orthopaedic resident trainees. This information is of importance as ChatGPT may have multiple future applications in revolutionizing orthopaedic residency physician education. For example, ChatGPT has the ability to provide instant feedback to residents; rather than waiting for a mentor or supervisor to review work, residents can ask ChatGPT questions and receive an answer immediately. Second, ChatGPT has the ability to simulate real-life scenarios. For example, residents can use ChatGPT to practice making surgical decisions in virtual cases. Lastly, ChatGPT has the potential to remain up-to-date with the latest developments in orthopaedic surgery by providing easy access to the latest research and guidelines, thereby helping residents to stay informed and improve the quality of care they provide to patients. However, the caveat and central paradigm to all of the aforementioned potential advantages is the accuracy and reliability of ChatGPT's responses, something that our study aims to elucidate and validate via standardized comparison with orthopaedic resident knowledge tested on the OITE.

Our aim was therefore to compare the performance of ChatGPT on the OITEs administered over the past five years versus local and national orthopaedic resident performance. By evaluating the performance of ChatGPT in this standardized exam, we are also testing the potential and utility of incorporating ChatGPT as a didactic component for medical training in this and other residency programs. We hypothesized that ChatGPT would be able to perform at the level of an orthopaedic residency trainee at both the local and national levels.

## Materials and methods

Question selection and processing

OITE questions were accessed from the American Academy of Orthopaedic Surgeons (AAOS) ResStudy online learning platform website (Rosemont, IL) for the 2018, 2019, 2020, 2021, and 2022 examinations [11]. A random number generator {randi([1 total number of questions], 10, 20)} was created on MATLAB (MathWorks®, Natick, Massachusetts, United States) to generate 200 questions in 10 sets of 20 for each examination year. Images without direct links from questions were uploaded to the image hosting service Imgur (MediaLab, Santa Monica, California, United States), which is specified as a readable weblink by the ChatGPT platform. All questions were entered into ChatGPT version 3.5 (OpenAI, San Francisco, California, United States) in a standardized format shown in Table 1 and exemplified in Table 2.

**Table 1:** Standardized question input methodology for OITE question text and images into ChatGPT Questions will either be purely text or incorporate a single or multiple images and videos. OITE: Orthopaedic In-Training Examination; AAOS: American Academy of Orthopaedic Surgeons

Body of question text, as exactly copied from the OITE in AAOS							
Image 1: (link), Image 2: (link),…							
Video 1: (link), Video 2: (link)							
(a) Answer choice 1							
(b) Answer choice 2							
(c) Answer choice 3							
(d) Answer choice 4							

**Table 2:** Example of test question input

Which of the following best describes how brace treatment of buckle fractures of the distal radius in pediatric patients compares with cast treatment?							
(a) Equivalent in terms of outcomes and pain control							
(b) Higher reinjury rate							
(c) More pain							
(d) More expensive							

**Figure 1:**
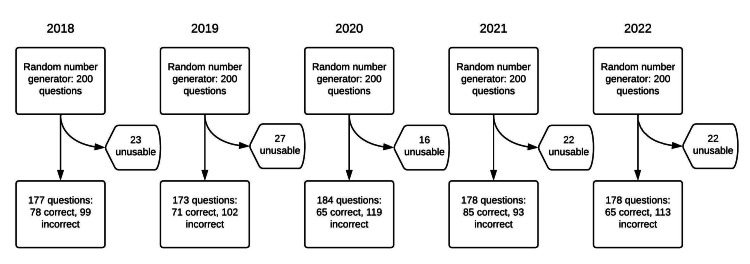
Study flowchart overview of OITE question selection and processing for ChatGPT OITE: Orthopaedic In-Training Examination

Questions resulting in refusal to answer after three separate input attempts, or generating ambiguous answers, or multiple answers, were designated unusable. A schematic overview of the selection process is provided (Figure 1). For the 2018 OITE, 200 out of 270 questions were randomly chosen, of which 23 were unusable, yielding 177 usable questions. For the 2019 OITE, 200 out of 260 questions were randomly chosen, of which 27 were unusable, with 173 usable questions. For the 2020 OITE, 200 out of 215 questions were randomly chosen, of which 16 were unusable, with 184 usable questions remaining. For the 2021 OITE, 200 out of 213 questions were randomly chosen, of which 22 were unusable, with 178 usable questions. For the 2022 OITE, 200 out of 207 questions were randomly chosen, of which 22 were unusable, with 178 usable questions.

Calculations

The mean percentage of correct ChatGPT responses for combined OITE years (2018, 2019, 2020, 2021, and 2022) was calculated by dividing the number of correct answers by the total number of answers, and multiplying by 100, after excluding all unusable questions. In order to calculate the percentage of correct responses for ChatGPT for each individual OITE year, for each set of 20 questions, we calculated the number of correct answers divided by the total answers, and multiplied by 100, after excluding all unusable questions. We then generated the average of the 10 sets of 20 questions for each respective OITE year.

Resident OITE performance data (national and institutional)

The annual AAOS "Orthopedic In-Training Technical Reports" for 2017, 2018, 2019, 2020, 2021, and 2022 provided summaries of the OITE results for all residents at Accreditation Council for Graduate Medical Education (ACGME)-accredited programs nationally in the United States [9]. We also utilized internal institutional OITE performance reports to obtain an anonymized summary of our local residency program performance over the same years. All data were used in a deidentified fashion and mined for descriptive statistics (mean and standard deviations), which were then used as a comparator versus ChatGPT performance.

Statistical analysis

All results were assessed for normality using the Kolmogorov-Smirnov test. As the data were not normally distributed, analyses were performed using the one-sample Wilcoxon test or the Kruskal-Wallis test with the Dunn-Sidak correction for multiple comparisons. All analyses were performed using MATLAB R2019a (MathWorks®, Natick, Massachusetts, United States) and GraphPad Prism 9.1.2 (GraphPad, La Jolla, California, United States). Graphs were generated using GraphPad Prism 9.1.2.

Power calculation

Assuming a standard power of 0.8, a mean and standard deviation of OITE performance of 63.15% and 8.79%, respectively (based on the 2021 performance of all ACGME-accredited programs), and a ChatGPT performance of 16.31% below resident performance (based on comparisons made between ChatGPT and 2017-2021 national resident OITE performance), a sample size of 5 is required [MATLAB >nout = sampsizepwr('t',[63.15 8.79],46.84,0.80)]. Our study was amply designed to achieve this desired sample size through the randomized sampling of five sets of five questions (a total of 25 questions) from OITE years 2017 to 2021.

Funding

This study was not supported by any external grant funding.

## Results

Overall, ChatGPT was provided 200 (10 sets of 20) randomly chosen questions from each OITE year (2018, 2019, 2020, 2021, and 2022). After elimination of unusable questions based on question selection and processing criteria, the usable questions' totals were 177 (2018), 173 (2019), 184 (2020), 178 (2021), and 178 (2022) (Appendix A). Examples of the input and responses done in ChatGPT are depicted in Appendix B.

Comparison of ChatGPT inter-year performance

**Figure 2:**
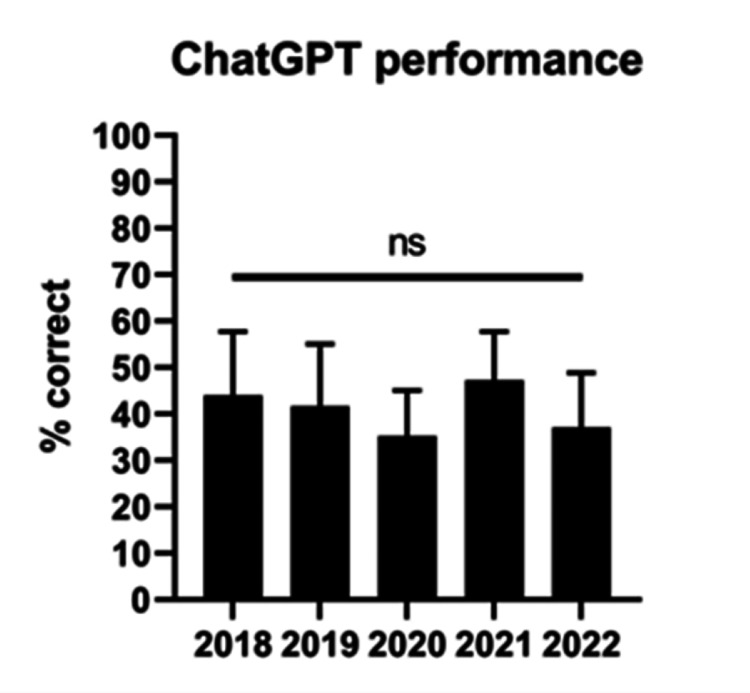
ChatGPT performance for each year n=10; ns: p>0.05. All comparisons done using the Kruskal-Wallis test with the Dunn-Sidak correction. Question number by year: 177 (2018), 173 (2019), 184 (2020), 178 (2021), and 178 (2022).

In terms of performance across the five years tested, ChatGPT scored the highest on the 2021 OITE (47.3%) and the lowest on the 2020 OITE (35.3%); however, performance over all five years was not significantly different in its own performance across the OITE years tested (p>0.05) (Figure 2).

Comparison of ChatGPT score and national average scores of orthopaedic surgery residents

**Figure 3:**
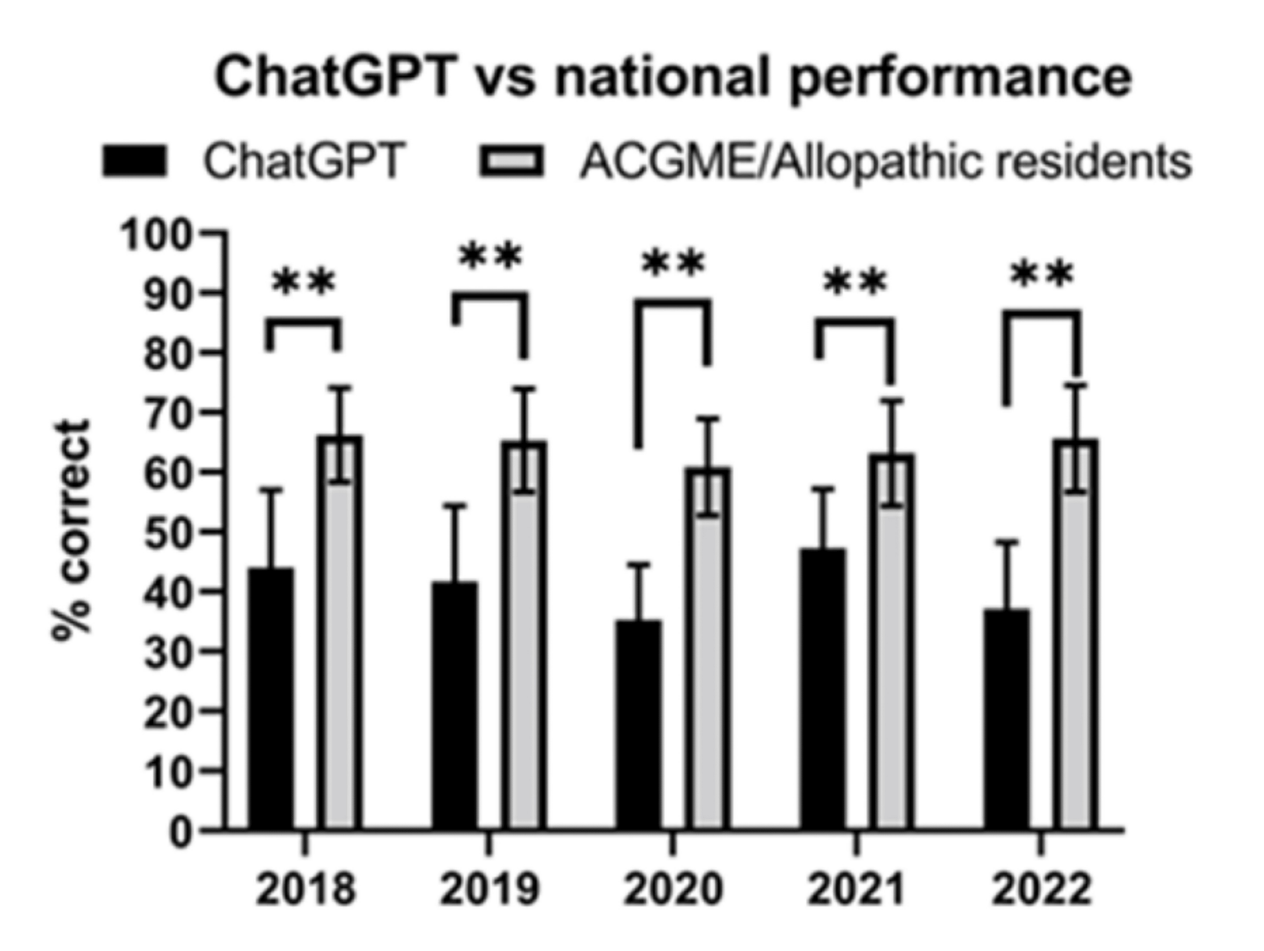
ChatGPT vs. national performance ChatGPT: n=10. ACGME/allopathic residents: n=3951 (2018), n=5110 (2019), n=4405 (2020), n=4466 (2021), and n=4496 (2022). **: p<0.01. All comparisons done using the one-sample Wilcoxon test, with the hypothetical mean being the ACGME/allopathic national performance. ACGME: Accreditation Council for Graduate Medical Education

**Figure 4:**
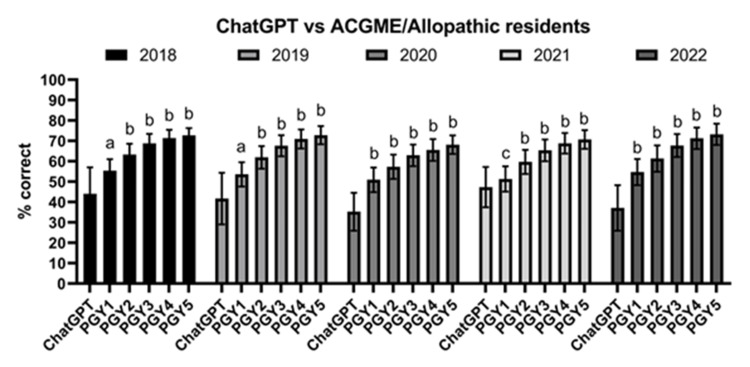
ChatGPT vs. ACGME/allopathic residents compared by training level ChatGPT: n=10. ACGME/allopathic residents: 2018: n=811 (PGY1); n=806 (PGY2); n=794 (PGY3); n=765 (PGY4); n=775 (PGY5). 2019: n=955 (PGY1); n=1040 (PGY2); n=1063 (PGY3); n=1075 (PGY4); n=977 (PGY5). 2020: n=855 (PGY1); n=915 (PGY2); n=895 (PGY3); n=881 (PGY4); n=861 (PGY5). 2021: n=879 (PGY1); n=911 (PGY2); n=900 (PGY3); n=895 (PGY4); n=881 (PGY5). 2022: n=897 (PGY1); n=910 (PGY2); n=906 (PGY3); n=889 (PGY4); n=893 (PGY5). a: p<0.05 compared with ChatGPT; b: p<0.01 compared with ChatGPT; c: p>0.05 compared with ChatGPT. All comparisons done using the one-sample Wilcoxon test, with the hypothetical mean being the ACGME/allopathic national performance. ACGME: Accreditation Council for Graduate Medical Education

ChatGPT significantly underperformed orthopaedic surgery resident national averages across all allopathic/ACGME-accredited orthopaedic surgery residents (Figure 3) (p<0.01). In a subgroup analysis for each year (2018-2022) (Figure 4), ChatGPT underperformed all PGY years (p<0.05 or p<0.01), with the exception of PGY1 in 2021 (p>0.05). 

Comparison of ChatGPT with local program residents

**Figure 5:**
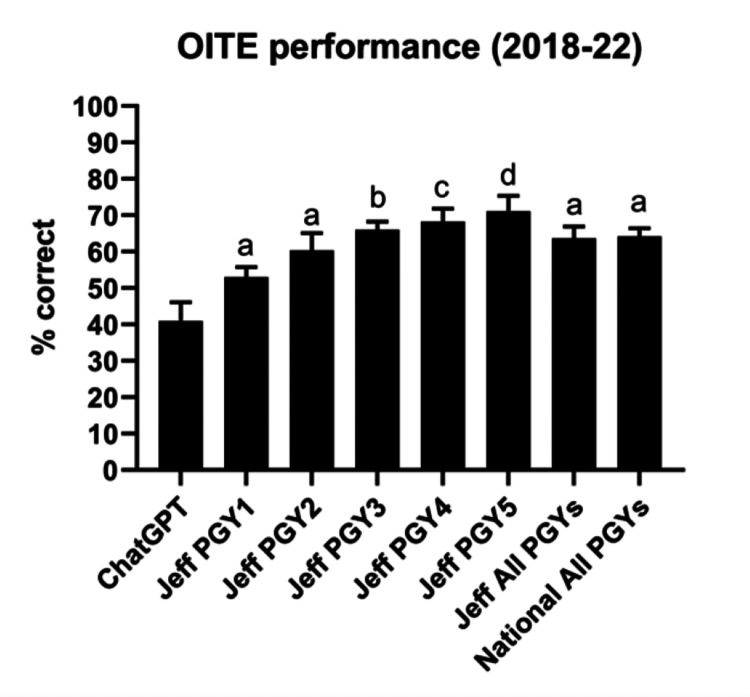
Performance of ChatGPT, Thomas Jefferson University residents, and all allopathic orthopaedic surgery residents Performance of ChatGPT, Thomas Jefferson University residents, and all (1) allopathic orthopaedic surgery residents nationally (2018, 2019) and (2) ACGME-accredited programs nationally (2020, 2021, 2022). n=5. a: p>0.05 when compared with ChatGPT; b: p<0.05 when compared with ChatGPT; c: p<0.01 when compared with ChatGPT; d: p<0.001 when compared with ChatGPT. All comparisons done using the Kruskal-Wallis test with the Dunn-Sidak correction. ACGME: Accreditation Council for Graduate Medical Education; OITE: Orthopaedic In-Training Examination

ChatGPT also underperformed all orthopaedic surgery PGY years at Thomas Jefferson University (Jeff) (Figure 5). However, this trend only reached significance on testing versus Jeff PGY3 (p=0.0444), Jeff PGY4 (p=0.0045), and Jeff PGY5 (p=0.0004) residents, who demonstrated significantly higher performance on testing. Jefferson resident performance overall increased with each PGY year for all five years tested.

## Discussion

This study aimed to assess the performance of ChatGPT on the OITE and compare its performance with local and national orthopaedic resident scores. The rationale for this endeavor was to elucidate the accuracy of recall of ChatGPT, as a surrogate for its utility as an educational adjunct for resident physicians, among other uses. We found that ChatGPT significantly underperformed on the OITE when compared to national resident averages across PGY1 to PGY5 levels, with the exception of PGY1 in 2021. The only statistically significant difference observed was in comparison with local institution PGY3, PGY4, and PGY5 resident averages. While it is groundbreaking for an automated, computer-based technology to perform at a statistically dissimilar level to orthopaedic residents who have trained for numerous years, the overall accuracy, particularly for image interpretation, appears to be lacking in this iteration as it is a language- and text-based tool. Nonetheless, implications for future resident teaching and clinical practice abound.

Prior literature has also demonstrated that ChatGPT is able to accomplish passing scores on advanced professional examinations [3,5]. For example, in one study evaluating ChatGPT's performance on USMLE examinations, questions were encoded as multiple-choice single-answer questions with no forced justification of positive and negative selections, exactly how the format is presented to test takers. This study by Kung et al. found that ChatGPT's accuracy for USMLE Steps 1, 2CK, and 3 was 55.1%, 56.9%, and 60.9%, respectively [5]. The limitation of this study was that the passing scores for this evaluation were only obtained after certain questions were censored to not include indeterminate responses. If all responses were included, then the scores for Steps 1, 2CK, and 3 would have been 36.1%, 56.9%, and 54.9%, respectively [5]. Our study differs as there is no censoring of indeterminate answers or of ChatGPT output in general. Another study, evaluating the performance of ChatGPT on a final examination given to Wharton MBA students on Operations Management, found that ChatGPT performed well on basic operations management and process analysis questions; however, it made mathematical errors at the level of sixth-grade math [3]. Overall, ChatGPT was able to receive a B to B- on the Wharton examination, which would be considered a passing grade in an upper-level MBA course [3].

In contrast to this, other studies have found that ChatGPT is not comparable to human performance. Two studies comparing the radiographic interpretations of ChatGPT to radiologists and trainees found ChatGPT to underperform across the board and often made blatant errors [12,13]. One study examining the performance of ChatGPT on the Bar exam found that though ChatGPT was able to achieve passing scores on two subsections, evidence and torts, it was not able to pass the entire exam. ChatGPT fell short of passing by approximately 17%, with an overall correct response rate of 53% on the MBE (multi-state bar examination) multiple-choice component [4]. The authors of this paper also conducted a sub-analysis, such that they found that ChatGPT's second-best answer for the incorrect questions was usually correct, indicating that it is possible that the design of the questions may be responsible for poor performance. Additionally, the bar exam is well known to be difficult with typically low passing rates, so this may be reflected in ChatGPT's performance as well [14].

The present study is not without limitations. First, while our study achieved the calculated sample size for the desired power, there is still the possibility of a type II error given the study design. Second, ChatGPT is a text-based LLM that is not currently validated for image interpretation. While the platform was able to take image inputs for the majority of questions (Figure 1), there were certain images that were misinterpreted or not readable by the software. These image-based questions were nonetheless included in our study for a realistic estimation of ChatGPT's performance. Our study also has notable strengths. First, it is the first study of its kind to evaluate the performance of ChatGPT on an annual OITE, providing, for the first time, insight into the performance capability of artificial intelligence (AI) on an advanced subspecialty training exam. Second, we ensured a consistent approach to randomization in question selection and data entry into ChatGPT. Third, analyses were conducted over multiple years of the OITE, with internal consistency demonstrated in findings.

The development of ChatGPT has the capability to dramatically influence medical education through instantaneous feedback and examination development. One could easily envision a future where AI tools such as ChatGPT could facilitate rapid responses to real-time clinical scenarios, offer radiographic interpretations, and apply the latest evidence-based treatment algorithms. It may also be used as a didactic tool and facilitate the question-explanation writing process for the development of future OITE or ABOS examination questions. However, the effect of AI on healthcare certainly does not end with education, as it also has the capability to be useful in documentation automation, electronic health record navigation [15], online patient support, even clinical decision-making [16-18], and disease prognostication [18].

## Conclusions

AI tools such as ChatGPT appear to have limitless potential but also unknown risks. Current concerns regarding the immediate use of this technology relate to response accuracy, transparency, bias, and data privacy. This study provides an early evaluation of ChatGPT's performance on the OITE in comparison to both national and institutional trainees. While the LLM scored lower on average than most trainees, one could predict that continual advancements in the field of AI will bridge this gap over time, the pace of which will only be enhanced as ChatGPT becomes more ubiquitous. With this in mind, ChatGPT will inevitably be used by trainees, not only in preparation for the OITE but also in other aspects of their education. Only time will tell when and how this powerful technology changes trainee education. One thing is clear however: appropriate evaluation and oversight are necessary to ensure these tools are leveraged properly.

## Figures and Tables

**Figure 6:**
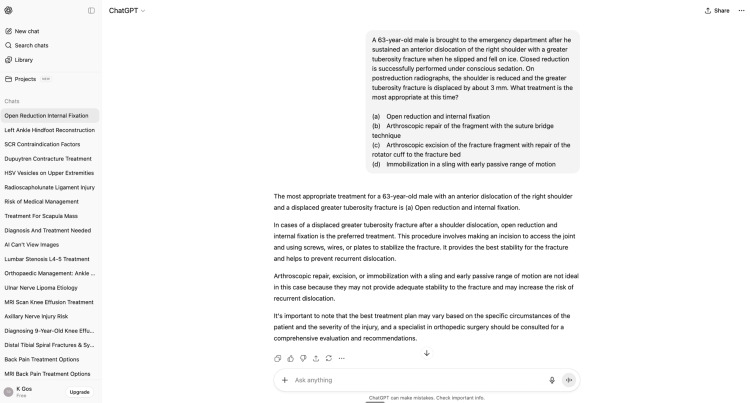
ChatGPT OITE question response 1 OITE: Orthopaedic In-Training Examination

**Figure 7:**
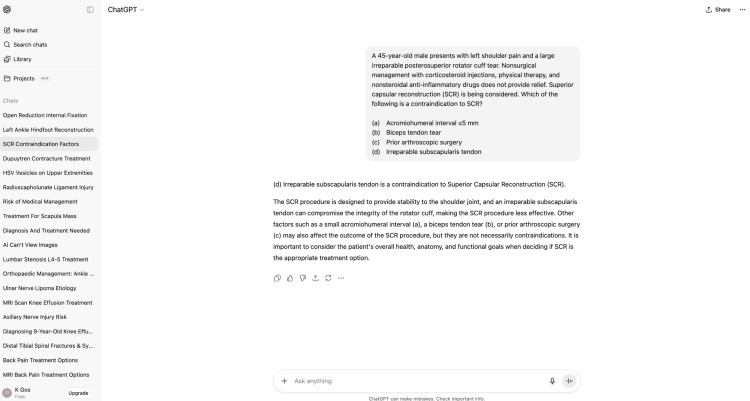
ChatGPT OITE question response 2 OITE: Orthopaedic In-Training Examination

**Figure 8:**
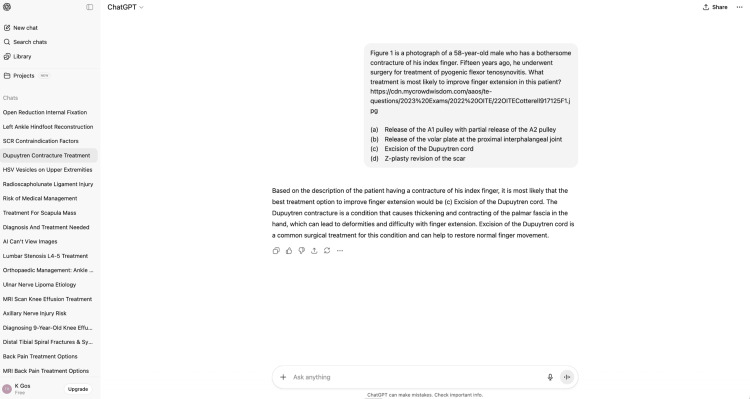
ChatGPT OITE question response 3 OITE: Orthopaedic In-Training Examination

**Figure 9:**
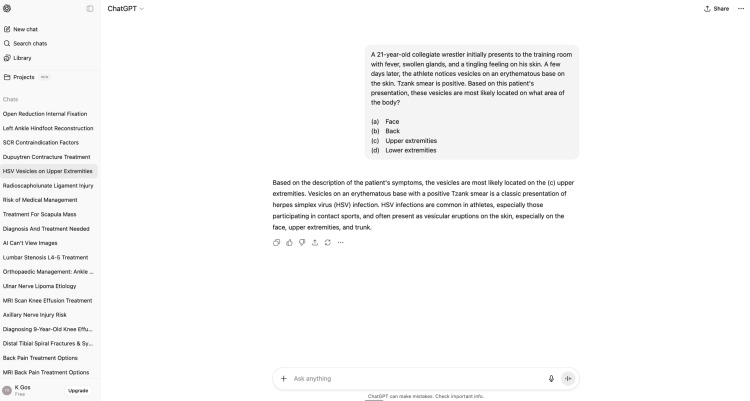
ChatGPT OITE question response 4 OITE: Orthopaedic In-Training Examination

## Appendices

Appendix A

**Table 3:** Total OITE items used in ChatGPT testing (2018-2022) OITE: Orthopaedic In-Training Examination

Year	Total OITE items	Randomly sampled	Unusable	Usable
2018	275	200	23	177
2019	275	200	27	173
2020	275	200	16	184
2021	275	200	22	178
2022	275	200	22	178

Appendix B
